# Proteome dataset of chili pepper plant (*Capsicum frutescens*) infested by broad mite *(Polyphagotarsonemus latus)*

**DOI:** 10.1016/j.dib.2021.107095

**Published:** 2021-04-25

**Authors:** Sachin S. Patavardhan, Kriti Awasthi, Suhasini Suresh, Pratigya Subba, Mohd Altaf Najar, Leo D'Souza, Shashi Kiran Nivas, Thottethodi Subrahmanya Keshava Prasad

**Affiliations:** a Laboratory of Applied Biology, St Aloysius College (Autonomous), Mangalore, India; b Center for Systems Biology and Molecular Medicine, Yenepoya Research Centre, Yenepoya (Deemed to be University), Mangalore, India; c Department of Biosciences, Mangalore University, Mangalore, India

**Keywords:** *Capsicum frutescens*, Mass spectrometry, Proteomics, Plant-pathogen interactions, Agriproteomics, Broad mite

## Abstract

The dataset presented in this article is associated with the TMT (Tandem mass tag) labeled proteomics of chili pepper plant (*Capsicum frutescens*) infested by a broad mite (*Polyphagotarsonemus latus*). Data was captured using a nano liquid chromatography system coupled with high-resolution Orbitrap FusionTribridmass spectrometer. Proteomics data was analyzed using the Proteome Discoverer version 2.4 tool using MASCOT and SequestHT algorithms. We have identified a total of 5,807 proteins supported by 48,555 unique peptides and 1,279,655 peptide-spectrum matches. Individually, 5,186 proteins were detected in healthy leaf samples, 5,193 in infested leaf sample, 5,194 proteins in healthy meristem sample, and 5,196 proteins in infested meristem samples. Datasets obtained from reciprocal blast against the *Arabidopsis thaliana* proteome database enabled the prediction of protein-protein interactions, and subcellular localization of differentially expressed proteins, which are also included in this article. Data presented in this article has been deposited in the ProteomeXchange Consortium via the PRIDE repository, which can be accessed through the accession ID: PXD018653.

## Specifications Table

**Table:** 

Subject	Plant Science
Specific subject area	Plant–insect interaction
Type of data	Table Figure
How data were acquired	Thermo Scientific Orbitrap Fusion Tribrid mass spectrometer (Thermo Fischer Scientific, Bremen, Germany) Proteome Discoverer 2.4with SequestHT and MASCOT (Matrix Science,London, UK; version 2.2)
Data format	Analyzed
Parameters for data collection	Sample collection was carried out from a broad mite-infested field. Plants grew at an ambient temperature of 27 °C with a relative humidity of 65–85%. Meristem and leaf samples of 12-weeks-old healthy and infected plants were collected for the proteomics analysis.
Description of data collection	The plant sample was homogenized using liquid nitrogen, proteins were extracted, disulphide bonds were reduced and alkylated. Proteins were subjected to trypsin digestion. Peptides were ladled with tandem mass tags (TMT) and fractionated using basic reverse-phase liquid chromatography (bRPLC). Peptide fractions were subjected to high-resolution mass spectrometry analysis to obtain peptide sequence information. Data was searched against *Capsicum annuum* (Pepper Zunla 1 Ref_v1.0) protein database to fetch peptide spectrum matches, from which a list of corresponding peptides and proteins were obtained. Identified proteins were subjected to bioinformatic analysis.
Data source location	Institution: St Aloysius College (Autonomous) City/Town/Region: Mangaluru, Karnataka state Country: India Latitude and longitude for collected samples/data:12°52′26.3″N 74°50′45.9″E, 28 September 2018
Data accessibility	Repository name: ProteomeXchange Data identification number: PXD018653 Direct URL to data: ftp://ftp.pride.ebi.ac.uk/pride/data/archive/2020/08/PXD018653 Or http://proteomecentral.proteomexchange.org/cgi/GetDataset?ID=PXD018653 Supplementary files can be accessed from: https://data.mendeley.com; DOI: http://dx.doi.org/10.17632/5j93jhjh6n.1
Related research article	Patavardhan, S. S., Subba, P., Najar, A., Awasthi, K., D'Souza, L., Prasad, T. S. K. and Nivas, S. K., 2020. Plant–Pathogen Interactions: Broad Mite (Polyphagotarsonemuslatus)-Induced Proteomic Changes in Chili Pepper Plant (Capsicum frutescens). *OMICS: A Journal of Integrative Biology*, 24(12), 714–725.

## Value of the Data

• This study provides insight into the comparative proteomic analysis of broad mite (*Polyphagotarsonemus latus*) infestation in chili (*Capsicum frutescens*)*.*
• The dataset describes the proteomic landscape of plant-mite interaction. These findings can broadly aid ecological and agri-proteomic research to prevent and manage pest-induced crop damage.
• Altered signaling pathways and hormone regulation mechanisms can be explored further to study the pest-induced plant hyper responses.
• The role of differentially expressed proteins in plant defense mechanisms can be assessed to design novel, eco-friendly molecules to deal with plant pathogens and post-infestation damage.

## 1 Data Description

**Fig. 1:**
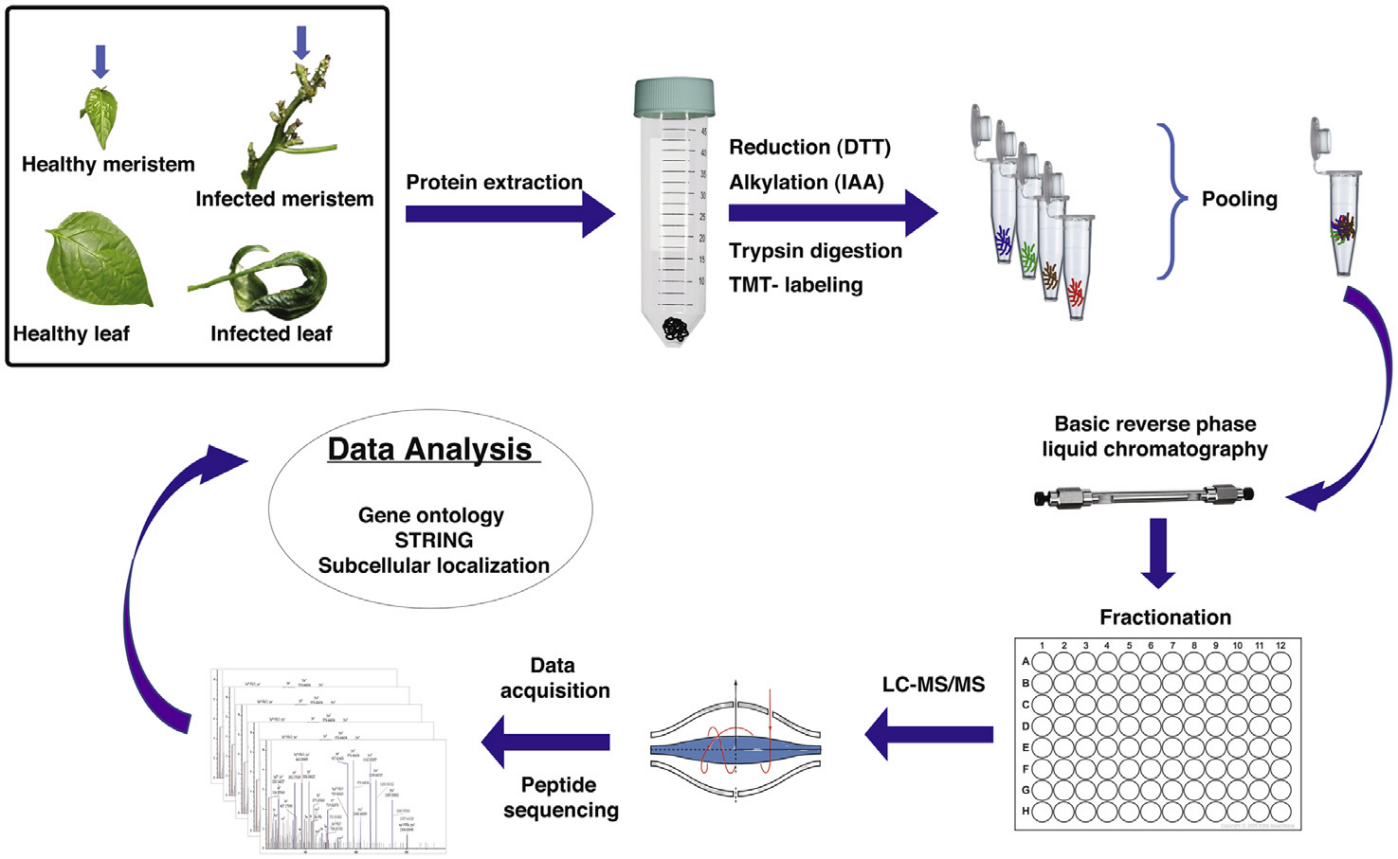
Representative experimental workflow of the dataset described in this study. Broad mite (*Polyphagotarsonemus latus*) infested birds eye chili (*Capsicum frutescens*) samples were collected and proteins were extracted, quantified using BCA assay. Trypsin digested peptides were labeled with tandem mass tags (TMT), subjected to basic reverse-phase liquid chromatography-based fractionization, and analyzed in a high-resolution orbitrap mass spectrometer. The acquired data is searched against *Capsicum annuum* (Pepper Zunla 1 Ref_v1.0) dataset using Sequest HT and MASCOT algorithms.

**Fig. 2:**
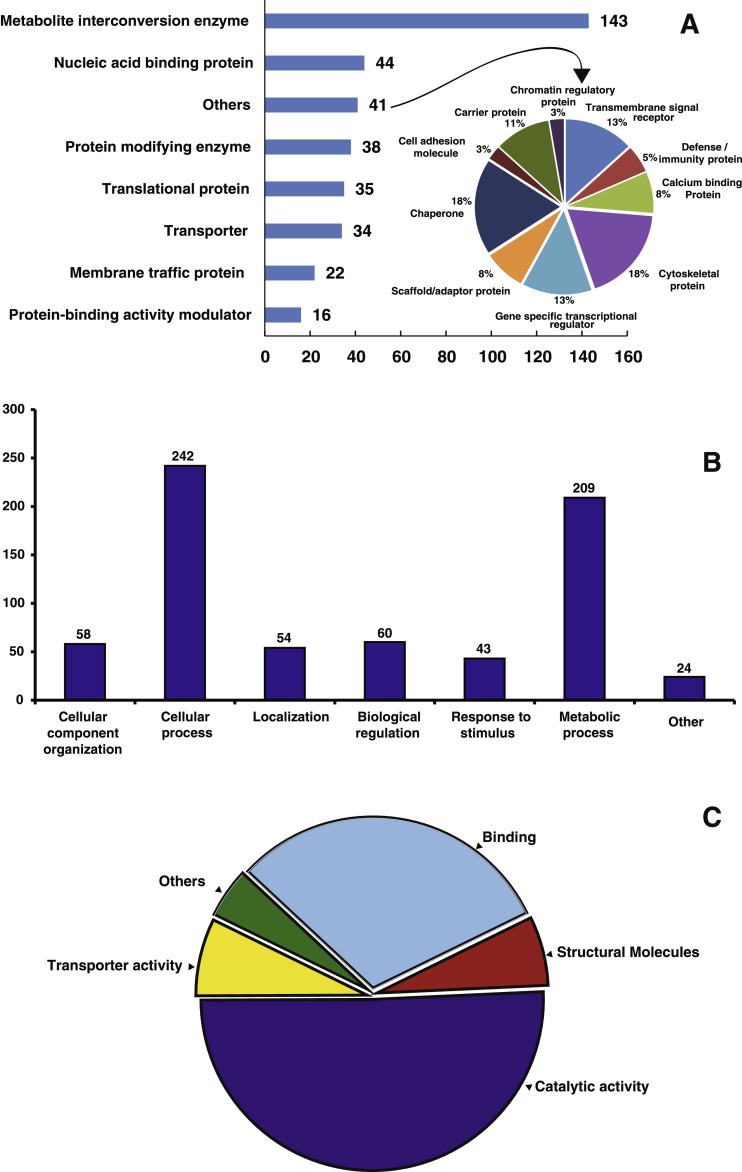
Gene Ontology and functional annotation of identified proteins of leaf and apical meristem tissues of *Capsicum frutescens* (A) Protein classes, (B) Biological process, (C) Molecular functions.

**Fig. 3:**
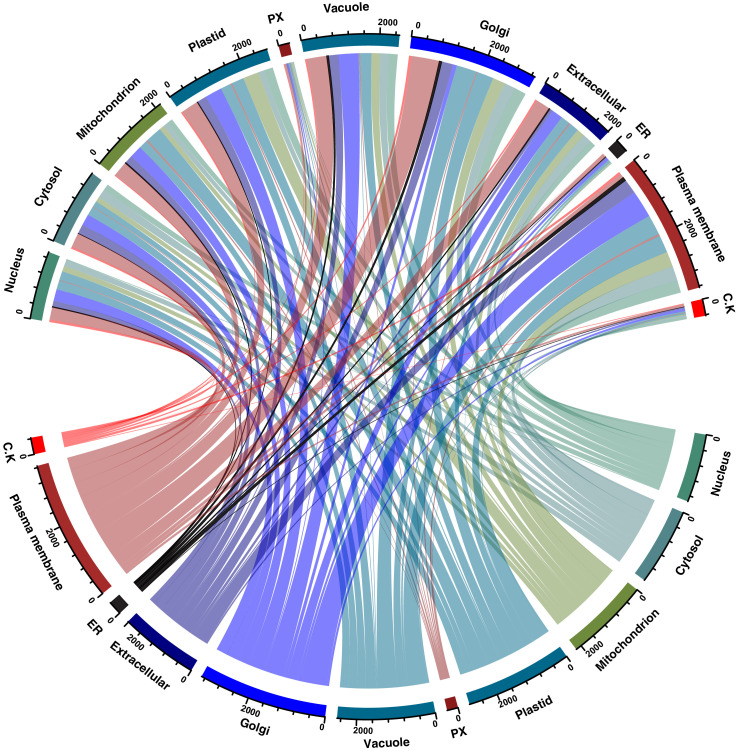
Chord diagram representing subcellular locations of identified proteins as predicted by SUBA4 tool. Only those proteins were selected which are previously reported in organelle-specific mass spectrometry-based proteomics experiments. Proteins that are present across different organelles are denoted by the colored connecting arc. The thickness of the arc corresponds to the number of proteins. Proteins that are exclusively present in an organelle, denoted by an arc that connects to the same organelle.

**Fig. 4:**
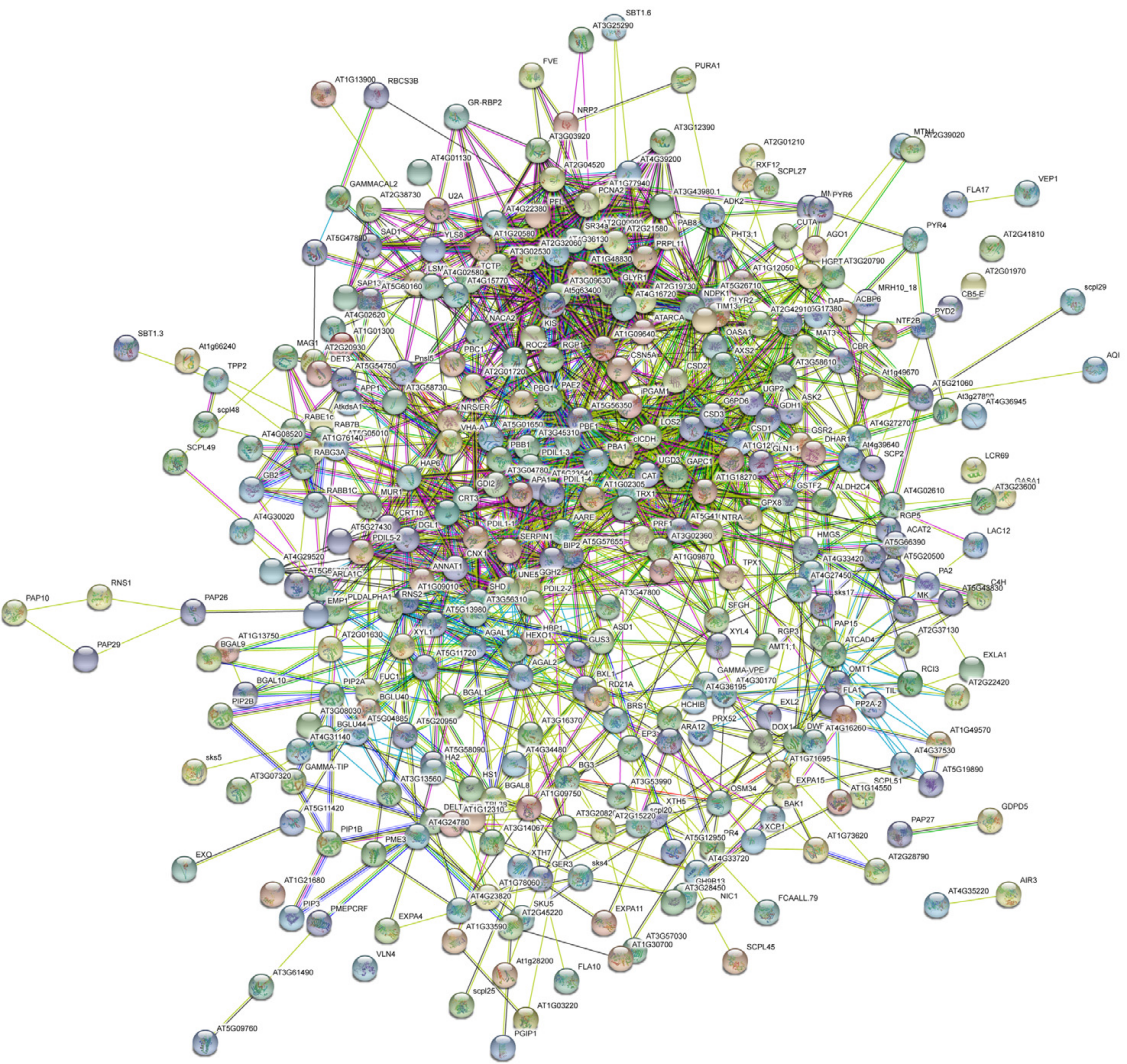
STRING Interaction network of extracellular proteins identified in the dataset, as annotated by SUBA4 tool.

This data set describes the proteins identified in *Capsicum frutescens* infected by a broad mite (*Polyphagotarsonemus latus*). Fig. 1 represents the overall pipeline of the experimental design. TMT-labeled shotgun proteomics analysis led to the identification of 5807 protein groups represented by 48,555 unique peptides and 1279,655 peptide-spectrum matches. Individually, 5186 proteins were detected in healthy leaf samples; 5193 in infested leaf samples; 5194 proteins in healthy meristems; and 5196 proteins in infested meristems. A list of all proteins including the differentially regulated proteins are presented in supplementary Table 1. Among the total proteins, we identified a large number of regulatory proteins such as kinases, phosphatases, transcription factors etc. The proteins categorized according to the activities are presented in supplementary Table 2. Data provided in this article were analyzed and represented in detail in the article, “Plant-Pathogen Interactions: Broad Mite (*Polyphagotarsonemus latus*)-Induced Proteomic Changes in Chili Pepper Plant (*Capsicum frutescens*)” [1]. Identified proteins were subjected to reciprocal BLAST(Basic Local Alignment Search Tool;blastp) against the *Arabidopsis thaliana* proteome to fetch the respective counterparts in *Arabidopsis thaliana* (supplementary Table 3). The Arabidopsis protein IDs were further analyzed by Gene Ontology analysis as represented in Fig. 2. The SUBA4 tool [2] was used to fetch the subcellular location of the proteins. Out of all identified proteins, the largest number of the proteins represented extracellular origin. Fig. 3 and supplementary Table 4 presents output data obtained from protein localization prediction [3]. Fig. 4 illustrates protein-protein interactions of extracellular proteins as predicted by STRING tool (Search Tool for the Retrieval of Interacting Genes/Proteins) [4]. The output data of STRING analysis is presented in Supplementary Table 5. The interactive network diagram was created using Cytoscape [5] and attached as supplementary file 6. List of identified capsicum proteins mapped to different KEGG Orthology and proteins mapped to KEGG pathways, KEGG brite, KEGG modules are presented in supplementary Table 7. A partial list of proteins identified in *Capsicum frutescens* leaf and meristem associated with plant-pathogen interaction is presented in Table 1. The data serves as a useful resource for scientists working on *Capsicum frutescens* and in the field of plant-pathogen interactions. Compared to our previously published article, here, we extended our analysis to all the proteins identified in this study to gather an overview of protein networks operative in *Capsicum frutescens* under control and infected conditions. The differentially regulated proteins identified may serve as targets that may be utilized in future to generate pathogen resistant crop plants.

**Table 1:** Partial list of proteins identified in *Capsicum frutescens* leaf and meristem associated with plant-pathogen interaction as annotated by KEGG pathways.

Protein accession	Name of the protein	Fold change in leaf	Fold change in meristem
XP_016537868.1	3-ketoacyl-coa synthase 10	1.12	0.45
XP_016538117.1	Mitogen-activated protein kinase homolog MMK2	1.88	1.37
XP_016539393.1	Basic form of pathogenesis-related protein 1-like	4.44	4.01
XP_016543739.1	Probable calcium-binding protein CML49	2.66	0.93
XP_016547276.1	Heat shock protein 90–5, chloroplastic	1.56	1.17
XP_016547536.1	Calmodulin-like	2.95	0.88
XP_016547769.1	Probable calcium-binding protein CML48	2.05	0.76
XP_016551150.1	PTI1-like tyrosine-protein kinase 1 isoform X1	1.68	0.92
XP_016551464.1	RPM1-interacting protein 4-like isoform X1	1.99	0.93
XP_016554291.1	Calcium-dependent protein kinase SK5-like	1.68	1.13
XP_016555153.1	Probable disease resistance protein At1g61190	3.92	1.49
XP_016555855.1	Calcium-binding allergen Ole e 8-like	1.76	0.95
XP_016562961.1	Glycerol kinase	1.58	1.53
XP_016567554.1	Mitogen-activated protein kinase 6	2.74	0.59
XP_016570057.1	Calcium-dependent protein kinase 11-like	1.71	1.20
XP_016578314.1	Protein EDS1-like	2.47	1.25
XP_016580574.1	Chitin elicitor receptor kinase 1-like isoform X1	3.30	1.44
XP_016573586.1	Pto-interacting protein 1-like	1.53	0.99
XP_016541443.1	Pathogenesis-related leaf protein 4-like	0.43	0.65
XP_016547427.1	Mitogen-activated protein kinase homolog NTF4	0.47	1.16
XP_016564510.1	Heat shock protein 83-like	0.64	1.31
XP_016565607.1	Elongation factor Tu, mitochondrial	0.65	0.86
XP_016565630.1	Calcium-dependent protein kinase 4	0.59	1.04
XP_016572842.1	Caltractin-like isoform X1	0.65	0.91
XP_016580498.1	Heat shock protein 90–6, mitochondrial	0.55	0.96

## 2 Experimental Design, Materials and Methods

### 2.1 Sample collection

Samples were collected from the broad mite-infested field. Plants grew at an ambient temperature of 27 °C, relative humidity was 65–85%. Leaf and apical meristems of healthy and infested plants were collected from 12 week old plant and snap-frozen in liquid nitrogen, stored at −80 °C until further analysis.

### 2.2 Protein extraction

Protein extraction was done according to [6]. one gram of the Samples were pulverized in liquid nitrogen using a mortar and pestle. Two grams of ground plant material was vortexed in pre-chilled 10 ml homogenization buffer containing 40% sucrose, 1 mM EDTA(ethylenediaminetetraacetic acid) (pH 7.5), 50 mM HEPES (4-(2-hydroxyethyl)−1-piperazineethanesulfonic acid)-KOH (potassium hydroxide)(pH 7.5), beta-mercaptoethanol (1%). 15 ml Tris equilibrated phenol was added and vortexed,this mixture was placed on a rocker at 4 °C for 15 min. Centrifugation was carried out at 5000 g for 15 min to separate the supernatant. Proteins were precipitated at −20 °C using 5 vol of 0.1 mM ammonium acetate in chilled methanol. Pellet was washed twice with 0.1 mM ammonium acetate in chilled methanol. Centrifugation was carried out to collect the protein pellet and washed with 80% acetone. Air-dried pellets were resuspended in a minimum amount of solubilizing buffer containing 2 M thiourea, 8 M urea, and CHAPS (2%) (3-[(3-*cholamidopropyl*) *dimethylammonio*]−1-propanesulfonate). Protein quantification was performed using Pierce™ BCA (Bicinchoninic acid) protein assay kit (Thermo Fisher Scientific, catalog number: 23,225) according to the manufacturer's protocol. Quality check was done by running SDS-PAGE (sodium dodecyl sulfate–polyacrylamide gel electrophoresis).

### 2.3 Trypsin digestion

Based on BCA assay, 100 µg protein in 600µL of Triethylammonium bicarbonate (TEABC) buffer were taken from each sample for reduction, alkalization and trypsin digestion. Disulfide bonds were reduced using 5 mM dithiothreitol (DTT) at 60 °C for 45 min, alkylated using 5 mM iodoacetamide (IAA) in dark at room temperature for 30 min. Samples were subjected to enzymatic digestion using sequencing grade TPCK (L-(tosylamido-2-phenyl) ethyl chloromethyl ketone) treated trypsin (trypsin: protein ratio of 1:20) overnight at 37 °C. Completion of trypsin digestion was checked by running SDS-PAGE of pre-digest and post-digest samples. Tryptic peptides were vacuum dried and resuspended in 50 mM TEABC buffer . TMT (Thermo Fisher Scientific, catalog number: 90,061) labeling was carried out as per the suppliers’ protocol. TMT-sixplex kit labels 126, 128, 129 and 130 were used for the infected leaf, infected meristem, healthy meristem and healthy leaf samples, respectively. 2 µg equivalent labeled peptides were pooled and analyzed in an orbitrap mass spectrometer to check the labeling efficiency. After confirmation, TMT-labeled peptides were pooled from all conditions. The pooled sample was fractionated using the basic reverse-phase liquid chromatography (bRPLC) into 96 fractions. These fractions were pooled and concatenated into 12 fractions. The separation was carried out in a XBridge C18, 4.6 × 250mmcolumn (Waters, Milford, USA; catalog number: 186,003,117). A gradient of 0–100% of solvent A (10 mM TEABC in the water at, pH 8.5) to solvent-B [90% ACN(Acetonitrile) in 10 mM TEABC] was used for 120 min. Separation was carried out using Hitachi's chromatography systems connected to UV- Visible detector set to 280 nm attached to a fraction collector (Gilson FC 204).

### 2.4 Desalting and cleanup

The fractionated peptides were desalted using SCX (strong cation exchange) stage tips (3M™ Empore™ Discs). STAGE (STopAnd Go Extraction) tips were prepared using 250µL pipette tips. Peptides were resuspended in trifluoracetic acid [TFA (0.1%)]. SCX columns were equilibrated using 70µL 70% ACN and thrice 70µL 0.1% TFA. Columns were loaded with peptides, flow-through is discarded. Peptides bound to the resin, are washed with 0.2% TFA. After washing, peptides were eluted out of the column using 70µL50mM of ammonium acetate in 50% acetonitrile and 0.5% formic acid.

### 2.5 Mass spectrometry analysis

After the label check run, pooled TMT labeled peptides were subjected to peptide sequencing using nano-LC-MS (Liquid chromatography-mass spectrometer) system Easy-nLC-1200 nanoflow liquid chromatography system (Thermo Fischer Scientific) connected to Orbitrap Fusion Tribrid mass spectrometer. Data were acquired using three technical replicates. Desalted pooled, labeled peptides were reconstituted in 0.1% formic acid and loaded onto a 2 cm trap column (NanoViper, 3 µm C18 Aq, Thermo Fisher Scientific). Peptides were separated using a 15 cm analytical column (NanoViper, 75 µm silica capillary, 2 µm C18 Aq) at a flow rate of 300 nL/min. Peptide separation was carried out in a gradient program 5–35%, for 120 min consisting of solvent A (0.1% formic acid) to solvent B (80% acetonitrile in 0.1% formic acid). Global MS survey scan range was set to 400–1600 *m/z* (120,000 mass resolution at 200 *m/z*) in a data-dependent mode using Orbitrap mass analyzer. The maximum injection time was 5 ms. Peptides with a charge state of 2–6 were considered for the analysis. The dynamic exclusion rate was set to 30 ms. For MS/MS analysis, data acquisition was carried out at top speed mode with 3 s cycles and subjected to higher collision energy dissociation with 34% normalized collision energy. MS/MS scans were carried out at a range of 100–1600 *m/z* using the Orbitrap mass analyzer at a resolution of 60,000 at 200 *m/z*. The maximum injection time was 120 ms. Internal calibration was carried out using a lock mass option (*m/z* 445.1200025) from ambient air.

### 2.6 Data analysis

Mass spectrometry derived raw data, were searched against the NCBI (National Center for Biotechnology Information) *Capsicum annuum* protein database(Pepper Zunla 1 Ref_v1.0)using Proteome Discoverer V2.4 platform (Thermo Scientific) with SequestHT and MASCOT algorithms to fetch protein lists. The search parameters were set as following: precursor mass tolerance was set to 20 ppm, fragment mass tolerance was set to 0.05 Da. TMT label at the peptide N terminus and Lys-residues, acetylation at the protein N terminus and oxidation of methionine were set as variable modifications. Carbamidomethylation of cysteine was set as a fixed modification. Other search parameters included two missed cleavages by trypsin and a 1% false discovery rate (FDR) maximum and minimum peptide length was set to 144 and 7, respectively. Since *Capsicum* sp. protein IDs are not supported by Gene Ontology and STRING analysis tools, the protein list obtained from the Proteome Discoverer platform was subjected to reciprocal BLAST (blastp) against the *Arabidopsis thaliana* data with the settings 60% identity and 80% query coverage to obtain the respective counterparts in *Arabidopsis thaliana* proteome. Gene Ontology analysis was carried out using PANTHER [7]. The protein-protein interaction analysis was carried out using STRING V.11.0 tool. Protein subcellular localization annotation was carried out using the SUBA4 tool. *Arabidopsis thaliana* protein IDs obtained from reciprocal BLAST were used as input to carry out data analysis. KEGG analysis was carried out using KEGG mapper version 4.3 [8]. Capsicum gene IDs were used to fetch KEGG pathways, KEGG Brite, and KEGG modules. Uniprot- Retrieve/ID mapping [9] and NCBI Datasets [10] tools were used to fetch corresponding capsicum ID's which were required for data analysis.

### 2.7 Data availability

The mass spectrometry raw data and the Proteome Discoverer output MSF files obtained from this study are publically available at ProteomeXchange Consortium (http://proteomecentral.proteomexchange.org) through the PRIDE partner repository (Project accession: PXD018653).

## Ethics statement

No human subjects or lab animals were used in the experiments; hence ethics committee approval was not required for this study. Supplementary files can be accessed from: https://data.mendeley.com; DOI: http://dx.doi.org/10.17632/5j93jhjh6n.1

## CRediT Author Statement

**Sachin S. Patavardhan:** Conceptualization, Methodology, Data Curation, Investigation, Formal Analysis, Writing - Original Draft, Visualization, Writing - Review & Editing, Project Administration, Software; **Kriti Awasthi:** Data Curation, Software. Suhasini Suresh: Formal Analysis; **Pratigya Subba:** Methodology, Writing - Review & Editing; **Mohd Altaf Najar:** Validation, Data Curation; **Leo D'Souza:** Funding Acquisition, Project Administration; **Shashi Kiran Nivas:** Conceptualization, Funding Acquisition, Project Administration, Supervision, Investigation, Review & Editing; **Thottethodi Subrahmanya Keshava Prasad:** Conceptualization, Methodology, Investigation, Software, Data Curation, Review & Editing.

## Declaration of Competing Interest

The authors declare that they have no known competing financial interests or personal relationships which have, or could be perceived to have, influenced the work reported in this article.

## Appendix Supplementary Materials

Image, application 1

Image, application 2

Image, application 3

Image, application 4

Image, application 5

Image, application 6

Image, application 7
